# Impact of systemic risk in the real estate sector on banking return

**DOI:** 10.1186/s40064-016-1693-8

**Published:** 2016-01-22

**Authors:** Shouwei Li, Qing Pan, Jianmin He

**Affiliations:** School of Economics and Management, Southeast University, Nanjing, 211189 China

**Keywords:** Systemic risk, Real estate, Banking return, Contingent claims analysis

## Abstract

In this paper, we measure systemic risk in the real estate sector based on contingent claims analysis, and then investigate its impact on banking return. Based on the data in China, we find that systemic risk in the real estate sector has a negative effect on banking return, but this effect is temporary; banking risk aversion and implicit interest expense have considerable impact on banking return.

## Background

The 2007–2009 financial crisis has shed light on the significance of systemic risk, and has made the concern about systemic risk increase. Most of the literature on systemic risk measures are about banking, as banks have long been known as a source of systemic risk. In fact, some researchers have provided evidence that the real estate sector is the most important source of systemic risk (Reinhart and Rogoff 2009; Allen and Carletti 2013; Ferrari and Pirovano 2014). However, studies on the real estate sector mainly focus on identifying the determinants of booms and busts in asset and/or real estate prices (Ferrari and Pirovano 2014).

Generally, bank portfolios have high exposure to the real estate sector directly or indirectly in many countries, such as the USA, Germany and some Asian countries (Martins et al. 2011). Distress in the real estate sector could affect the value of both the direct exposures in property loans and the real estate collaterals of loans, therefore, banks’ performance or risk could change significantly in the case of real estate sector collapse (Wheaton 1999).

This study aims to measure systemic risk in the real estate sector and to investigate its effect on banking return. It contributes to the research on the relationship between the real estate sector and banking sector by investigating the measure of systemic risk in the real estate sector based on the Contingent Claims Analysis, and by analyzing the impact of the real estate sector on banking return from the perspective of systemic risk. Besides, this paper empirically analyzes the Chinese real estate sector and the banking sector.

The paper is structured as follows. After this introduction, “Literature review” section presents a detailed literature review. “Methodology and data” section presents the methodology and the data. “Empirical results” section provides the main results, and “Conclusion” section puts forward a conclusion.

## Literature review

There is a growing literature on systemic risk, which is mainly about the three aspects of systemic risk: its definition (e.g., Bartholomew and Whalen 1995; Acharya 2009; Martínez-Jaramillo et al. 2010), factors that may cause changes in the level of systemic risk, and the measurement of systemic risk (Bisias et al. 2012). Factors that may alter the level of systemic risk include financial system consolidation (De Nicolo and Kwast 2002; Weiß et al. 2014), network structures (Nier et al. 2007; Lenzu and Tedeschi 2012; Chen and He 2012; Hautsch et al. 2015; Chen et al. 2015), the opaque (Jones et al. 2012) and hedge funds (Kambhu et al. 2007; Bianchi and Drew 2010). Most of the literature on systemic risk is about banking, while there is little research on systemic risk in the real estate sector. A rare example is that Meng et al. (2014) investigate the systemic risk and spatiotemporal dynamics of the US housing market at the state level based on the Random Matrix Theory.

As for risk in real estate sectors, scholars mainly investigate volatility of real estate sectors. For example, Crawford and Fratantoni (2003) employ three different types of models to forecast real estate volatility for five states: California, Florida, Massachusetts, Ohio, and Texas. Mi et al. (2014) develop a real estate volatility index based on Real Estate Investment Trusts over an extensive period of 1996–2012. Zheng (2015) measures housing price volatility by the conditional variance of a Generalized Auto Regressive Conditional Heteroscedasticity model under the Adaptive Expectations framework.

Since real estate often constitutes a major item on bank balance sheets, there is a lot of literature on the analysis of the relationship between the real estate sector and the banking sector from different perspectives, such as real estate price and bank lending (Park et al. 2010; Hott 2011; Arestis and González 2014), real estate price and bank stability (Koetter and Poghosyan 2010; Pan and Wang 2013), contagion risk between real estate sector and banking sector (Pais and Stork 2011), the impact of real estate risk on bank stock returns (Elyasiani et al. 2010; Martins et al. 2011), and so on.

In summary, there is a lot of literature concerned with the volatility of the real estate sector, while little attention has been paid to systemic risk measure in the real estate sector. Real estate often constitutes a major item on bank balance sheets, and affects banking return (Mei and Anthony 1995; He 2002; Elyasiani et al. 2010; Martins et al. 2011). However, according to our knowledge, there is no study on the investigation of the impact of the real estate sector on banking return from the perspective of systemic risk.

## Methodology and data

### Methodology

The approach of contingent claims analysis (CCA) is a framework that combines market-based and balance sheet information to obtain a comprehensive set of financial risk indicators (Saldías 2013). Recently it has been implemented to analyze banking systemic risk based on aggregated Distance-to-Default (DD) series (Saldías 2013; Harada et al. 2013; Singh et al. 2014). In this paper, we adopt the Weighted Average Distance-to-Default (*WADD*) series to measure systemic risk in the real estate sector. *WADD*_*t*_ is represented in the following equation.

1 $$WADD_{t} = w_{it} \sum\limits_{i = 1}^{N} {DD_{it} } ,$$

where *N* is the number of real estate firms, and *w*_*it*_ is the individual market capital weight.

In Eq. (1), *DD*_*it*_, the Distance-to-Default of the real estate firm *i* at time *t*, is calculated as

2 $$DD_{it} = \frac{{A_{it} - D_{it} }}{{A_{it} \sigma_{it} }},$$

where *A* is the value of firm assets, *D* the face value of firm debt, and $\sigma$ the volatility of firm assets. However, the value and the volatility of firm assets are unobservable. Based on the Black–Scholes model, we can obtain the following equations to calculate *A* and $\sigma$.

3 $$E_{it} = A_{it} N(d_{1} ) - D_{it} e^{ - rT} N\left( {d_{1} - \sigma_{it} \sqrt T } \right),$$

4 $$d_{1} = \frac{{\ln \left( {A_{it} /D_{it} } \right) + \left( {r + \sigma_{it}^{2} /2} \right)T}}{{\sigma_{it} \sqrt T }},$$

5 $$\hat{\sigma }_{it} = \frac{{A_{it} }}{{E_{it} }}N(d_{1} )\sigma_{it} ,$$

where *T* is the time horizon of debt, *r* the risk-free rate, *E* the market value of firm equity capital, $\hat{\sigma }$ the volatility of firm equity capital, and *N(.)* the cumulative normal distribution.

### Data

Generally, indicators, such as banks’ net interest margin (*NIM*), return on equity (*ROE*), return on assets (*ROA*) and stock return, are used to measure banking return (García-Herrero et al. 2009; Alessandri and Nelson 2015). China’s banking system is highly dependent on the deposit-loan interest margin, with interest margin being the main profit of banks. Therefore, we use the average of banks’ net interest margin (*NIM*) to measure banking return. Some studies have found that degree of risk aversion (*RA*) and implicit interest expense (*IIE*) are the determinants of banking return (Ho and Saunders 1981; Maudos and De Guevara 2004; Mensah and Abor 2014). Therefore, *RA* and *IIE* are chosen as control variables when conducting empirical analysis. Maudos and De Guevara (2004) find that the more banks’ own capitals are, the higher the degree of banks’ risk aversion is. Hence, we use the average of all banks’ proportions of equity capital to total assets to measure banking *RA*. Following Angbazo (1997), Saunders and Schumacher (2000) and Maudos and De Guevara (2004), we adopt the average of all banks’ proportions of the difference between non-interest expense and non-interest income to total assets to measure banking *IIE*.

There are 143 listed real estate firms in China. Considering the difference of the listed time, in this paper we analyze 97 listed real estate firms. There are only 16 listed banks in China. Besides, the net profit of the banking sector in China in 2014 is 15,500 million RMB, among which the net profit of the 16 listed banks is 12,500 million RMB. Therefore, the 16 listed banks can be a good representation for the banking sector. Stock codes of the 97 listed real estate firms and the 16 listed banks are listed in Table 1. Data employed in this paper stem from the Bankscope database, the Wind database and the quarterly reports of firms and banks, with the Bankscope database being the most comprehensive global database of banks, financial statements, ratings and intelligence, and the Wind database being a leading integrated service provider of financial data in China. The time interval is from the third quarter of 2007 to the second quarter of 2014. Following Romero et al. (2013) and Milne (2014), we set *T* be one year, *D* the face value of short-term liabilities plus half of that of long-term liabilities, and we calculate $\hat{\sigma }$ based on the standard deviation of daily equity logarithmic returns. Since what we can obtain is only the date of banks’ total liabilities, we set *D* be the total liabilities for banks. *r* is set as the one-year deposit interest rate during the trading period.

**Table 1:** List of real estate firms and banks

Stock code	Stock code	Stock code	Stock code	Stock code	Stock code	Stock code	Stock code
600007.SH	600555.SH	601588.SH	000567.SZ	600048.SH	600565.SH	900950.SH	000573.SZ
600052.SH	600568.SH	900955.SH	000608.SZ	600067.SH	600620.SH	000002.SZ	000609.SZ
600077.SH	600621.SH	000006.SZ	000616.SZ	600095.SH	600622.SH	000011.SZ	000667.SZ
600113.SH	600638.SH	000014.SZ	000718.SZ	600149.SH	600641.SH	000024.SZ	000797.SZ
600158.SH	600647.SH	000029.SZ	000803.SZ	600159.SH	600649.SH	000031.SZ	000809.SZ
600162.SH	600665.SH	000036.SZ	000838.SZ	600173.SH	600683.SH	000040.SZ	000886.SZ
600175.SH	600684.SH	000042.SZ	000897.SZ	600185.SH	600696.SH	000043.SZ	000909.SZ
600208.SH	600724.SH	000046.SZ	000926.SZ	600240.SH	600732.SH	000056.SZ	000965.SZ
600246.SH	600733.SH	000069.SZ	002016.SZ	600266.SH	600736.SH	000090.SZ	002077.SZ
600322.SH	600748.SH	000150.SZ	200011.SZ	600325.SH	600767.SH	000402.SZ	200024.SZ
600340.SH	600773.SH	000514.SZ	200029.SZ	600376.SH	600777.SH	000534.SZ	200056.SZ
600383.SH	600791.SH	000537.SZ	600393.SH	600823.SH	000540.SZ	600533.SH	600890.SH
000558.SZ	600000.SH	600015.SH	600016.SH	600036.SH	601009.SH	601166.SH	601169.SH
601288.SH	601328.SH	601398.SH	601818.SH	601939.SH	601988.SH	601998.SH	000001.SZ
002142.SZ							

## Empirical results

**Fig. 1:**
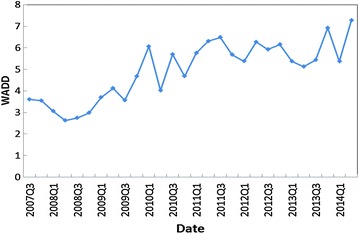
*WADD* of the real estate sector from the third quarter of 2007 to the second quarter of 2014

Figure 1 shows the result of *WADD* of Chinese real estate sector, and Table 2 presents the description statistics of the variables. We first test whether *WADD* is related to *NIM* based on Granger causality tests. Granger (1969) defines causality between two variables in terms of predictability. The causal relationship between two variables can be tested within a VAR framework, where the null hypothesis of no causality is tested via the significant contribution that past values of one variable can offer in predicting current values of another (Dergiades et al. 2013). Before conducting Granger causality tests, we apply Augmented Dickey–Fuller test (ADF) to *WADD*, *NIM*, *RA* and *IIE*. Based on the ADF statistic (Dickey and Fuller 1981), we test the null hypothesis for the existence of a unit-root against the alternative hypothesis of stationary variables, where the automatic selection of lags is based on Schwarz Information Criterion. The test results are shown in Table 2, from which we can see that the four variables are stationary, and are thus suitable for further Granger causality tests.

**Table 2:** Descriptive statistics and ADF tests

	WADD	NIM	RA	IIE
Mean	4.9554	2.5846	0.0583	0.0036
Max.	7.2809	3.1500	0.0631	0.0146
Min.	2.6368	2.2200	0.0498	0.0008
SD	1.3331	0.2530	0.0037	0.0027
ADF-statistic	−3.9518 (0.0234)	−3.4360 (0.0692)	−5.6367 (0.0009)	−3.7060 (−0.0393)

The numbers in parentheses refer to the p values

In Table 3, we report the result of the Granger causality test in VAR Framework. It can be seen from Table 2 that *WADD*, *IIE* and *RA* Granger cause *NIM* at the 1 % level of significance. This finding suggests that *WADD*, *IIE* and *RA* may be the factors that determine *NIM*. To investigate the impact of systemic risk in the real estate sector on banking return, we specify and estimate a vector-autoregressive (VAR) model. The VAR model is originally advocated by Sims (1980) as an alternative to simultaneous equation models. According the result of the Granger causality test, we estimate our VAR model with the four endogenous variables (*NIM, WADD, IIE* and *RA*). Based on Akaike information criterion, we set the lag of the VAR model to be 2. Table 4 shows the estimation results of VAR model. In addition, we find that the VAR model satisfies the stability condition, because no root lies outside the unit circle.

**Table 3:** Causality test results

Dependent variable: NIM			
Excluded	Chi-sq	df	Prob.
WADD	16.54417	2	0.0003
IIE	24.43692	2	0.0000
RA	18.24806	2	0.0001
All	32.07118	6	0.0000

**Table 4:** Estimation results of vector autoregression

	WADD	NIM	RA	IIP
WADD(−1)	−0.036288	0.105734	0.001240	0.000312
	(0.23098)	(0.03564)	(0.00061)	(0.00032)
	[−0.15711]	[2.96710]	[2.01734]	[0.96334]
WADD(−2)	0.353715	0.055955	0.000606	−0.000110
	(0.21662)	(0.03342)	(0.00058)	(0.00030)
	[1.63286]	[1.67426]	[1.05173]	[−0.36129]
NIM(−1)	−0.245814	0.419979	0.005668	0.006656
	(1.26348)	(0.19493)	(0.00336)	(0.00177)
	[−0.19455]	[2.15453]	[1.68521]	[3.76257]
NIM(−2)	−1.033027	−0.082488	−0.003100	−0.001881
	(1.38455)	(0.21361)	(0.00369)	(0.00194)
	[−0.74611]	[−0.38617]	[−0.84112]	[−0.97044]
RA(−1)	93.17694	−41.15077	−0.092349	−0.736102
	(65.3747)	(10.0860)	(0.17403)	(0.09153)
	[1.42528]	[−4.08001]	[−0.53065]	[−8.04234]
RA(−2)	−26.16846	37.18016	0.714498	0.527164
	(86.1895)	(13.2973)	(0.22944)	(0.12067)
	[−0.30362]	[2.79608]	[3.11410]	[4.36863]
IIP(−1)	−197.1447	101.6222	0.967578	1.047774
	(145.312)	(22.4186)	(0.38683)	(0.20345)
	[−1.35670]	[4.53293]	[2.50132]	[5.15015]
IIP(−2)	−44.92863	16.35838	−0.172899	−0.462508
	(113.633)	(17.5311)	(0.30249)	(0.15909)
	[−0.39539]	[0.93310]	[−0.57158]	[−2.90717]
C	3.827766	0.704900	0.003853	0.000238
	(3.36996)	(0.51991)	(0.00897)	(0.00472)
	[1.13585]	[1.35580]	[0.42955]	[0.05040]
R-squared	0.752010	0.838717	0.732881	0.890176
Adj. R-squared	0.635308	0.762820	0.607179	0.838495

Standard errors are in ( ) and t-statistics in [ ]

**Fig. 2:**
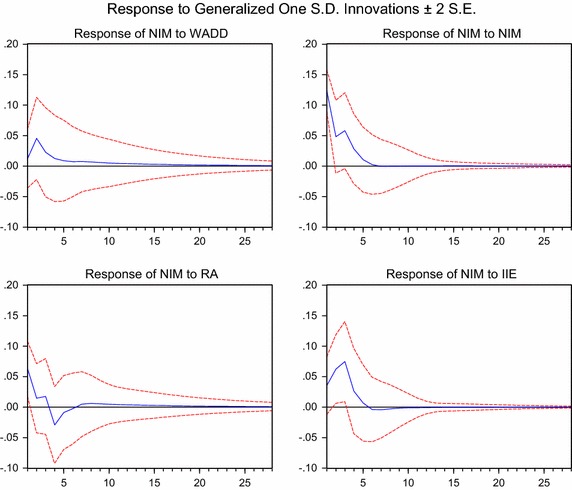
Responses of *NIM* to *WADD, NIM, RA* and *IIE*

Since it is infeasible to interpret estimated VAR-coefficients directly, researchers use the estimated coefficients to calculate impulse response functions (Trusov et al. 2009). Impulse response analysis enables us to examine the effect of a shock on various variables in a system with the change of time. Therefore, to better understand how shocks in the real estate sector are transmitted to the banking sector, the impulse response function of the VAR system is examined. Figure 2 shows how *NIM* responds dynamically to one-standard-deviation shocks to the variables in the VAR model. The response of *NIM* to *WADD* shock is positive, and it is the same as that of *NIM* to *NIM* shock. The response of *NIM* to *RA* shock is positive first and negative afterwards. After reaching to the lowest point, the response of *NIM* to *RA* shock gradually become positive, and then descends gradually to 0. The response of *NIM* to *IIE* shock is positive first and negative afterwards, and then descends gradually to 0.

The response of *NIM* to *WADD* shock is only 0.01 in the current period, but it increases over time and reaches 0.05 at the first quarter. Then, it descends gradually to 0 near the 10th quarter. After five quarters, *NIM* increases cumulatively by about 12.5 % caused by *WADD* shock. The effect direction of *RA* shock has changed from the 3*rd* quarter to the 4th quarter. The response of *NIM* to *RA* shock has declined persistently until reaching the lowest point −0.03 at the 4th quarter. Compared with *WADD* and *RA*, *IIE* has a more significant impact on *NIM*. The response of *NIM* to *IIE* shock gets to the maximum 0.075 at the 3*rd* quarter and falls quickly thereafter.

The results indicate that *WADD* has a positive effect on *NIM*. Note that the bigger *WADD* is, the smaller systemic risk in the real estate sector is. Therefore, banking return will decline when systemic risk in the real estate sector increases. Nevertheless, this kind of external effects won’t last for a long time, and are weaker than *RA* and *IIE* within banking systems.

## Conclusion

Based on Contingent Claims Analysis approach, we apply the weighted average Distance-to-Default series as systemic risk measure tools for the real estate sector. And then we use the vector autoregression model to investigate the impact of systemic risk on banking return in the real estate sector. Empirical results based on Chinese data shows that systemic risk in the real estate sector has a negative effect on banking return, but this effect is temporary. In addition, the degree of risk aversion and implicit interest expense also have considerable impact on banking return. Our findings have policy and practical implications. First, risk management and policies on the control and management of systemic risk in the real estate sector should not be ignored. Second, the exposition of banks to the real estate sector should be monitored. Finally, the underlying bank return generating models should incorporate an additional risk factor: systemic risk in the real estate sector. In this paper, we use banks’ net interest margin to measure banking return. However, there are many other indicators used in measuring banking return, such as stock return, return on equity and return on assets. Therefore, in the future, we would analyze the impact of systemic risk in the real estate sector on these indicators.
